# Bioinformatic Analysis of Plasma Apolipoproteins A-I and A-II Revealed Unique Features of A-I/A-II HDL Particles in Human Plasma

**DOI:** 10.1038/srep31532

**Published:** 2016-08-16

**Authors:** Toshimi Kido, Hideaki Kurata, Kazuo Kondo, Hiroshige Itakura, Mitsuyo Okazaki, Takeyoshi Urata, Shinji Yokoyama

**Affiliations:** 1 Institute of Environmental Science of Human Life, Ochanomizu University, Bunkyo-ku, Tokyo 112-8610, Japan; 2 Division of Diabetes, Metabolism and Endocrinology, Department of Internal Medicine, The Jikei University School of Medicine, Nishi-Shimbashi, Minato-ku, Tokyo, 105-8461, Japan; 3 Department of Food and Nutritional Science, Toyo University, Itakura-machi, Ora-gun, Gunma 374-0193, Japan; 4 Shinagawa East One Medical Clinic, Minato-ku, Tokyo 108-0075, Japan; 5 Tokyo Medical and Dental University, Bunkyo-ku, Tokyo 113-8519, Japan; 6 International Mibyou (Pre Symptomatic Medicine) Medical Center, Sanuki-chou, Ryugasaki, Ibaraki 301-0033, Japan; 7 Department of Pharmacogenomics, Showa University, Hatanodai, Shinagawa-ku, Tokyo 142-8555, Japan; 8 Nutritional Health Science Research Center, Chubu University, Matsumoto-cho, Kasugai 487-8501, Japan

## Abstract

Plasma concentration of apoA-I, apoA-II and apoA-II-unassociated apoA-I was analyzed in 314 Japanese subjects (177 males and 137 females), including one (male) homozygote and 37 (20 males and 17 females) heterozygotes of genetic CETP deficiency. ApoA-I unassociated with apoA-II markedly and linearly increased with HDL-cholesterol, while apoA-II increased only very slightly and the ratio of apoA-II-associated apoA-I to apoA-II stayed constant at 2 in molar ratio throughout the increase of HDL-cholesterol, among the wild type and heterozygous CETP deficiency. Thus, overall HDL concentration almost exclusively depends on HDL with apoA-I without apoA-II (LpAI) while concentration of HDL containing apoA-I and apoA-II (LpAI:AII) is constant having a fixed molar ratio of 2 : 1 regardless of total HDL and apoA-I concentration. Distribution of apoA-I between LpAI and LpAI:AII is consistent with a model of statistical partitioning regardless of sex and CETP genotype. The analysis also indicated that LpA-I accommodates on average 4 apoA-I molecules and has a clearance rate indistinguishable from LpAI:AII. Independent evidence indicated LpAI:A-II has a diameter 20% smaller than LpAI, consistent with a model having two apoA-I and one apoA-II. The functional contribution of these particles is to be investigated.

High density lipoproteins (HDL) in human plasma are relatively small and mostly spherical lipid-protein complex particles, having diameters 10–15 nm being composed of several hundred lipid molecules and a few apolipoprotein (apo) molecules^1^, mainly apoA-I and apoA-II^2^. HDL plays a key role in transport of cholesterol from the tissues to the liver for excretion into the bile either as cholesterol itself or after conversion to bile acids. The basic structure of HDL is considered to be a microemulsion with core lipids of esterified cholesterol and a small amount of triglyceride surrounded with surface components of phospholipids, unesterified cholesterol and helical amphiphilic apolipoproteins^1^. HDL is however structurally heterogeneous in size, density and chemical composition^3^. HDLs are metabolically active and therefore unstable particles that undergo enzymatic reactions, transfer/exchange of component molecules and accordingly remodeling in plasma as they carry cholesterol^4^.

ApoA-I is a predominant protein component of HDL along with a secondary dominant apoA-II^5^,^6^,^7^. Because HDL is a small particle that will accommodate only a few protein molecules per particle, apoA-II cannot be present in all HDL particles so that some of the HDL particles inevitably contain only apoA-I^2^. Accordingly, HDL particles can largely be classified into two categories; those associated only with apoA-I (LpAI) and those with both apoA-I and apoA-II (LpAI:AII)^5^,^6^,^8^. Human apoA-I is a single peptide of 243 amino acid residues^9^ while apoA-II is a disulfide-linked homodimer of 77-residue peptides^10^. Both have several helical segments that are believed responsible for their reversible binding to lipoprotein surface, enhance cholesteryl ester transfer protein reaction^11^ and generate HDL from cellular lipid in the presence of ABCA1^12^. Granted that distribution of the two apolipoproteins are merely statistical, these two types of particles may substantially be different in their properties and functions. The major factors for this differentiation may be 1) very poor potency of apoA-II to “activate” lecithin: cholesterol acyltransferase (LCAT) in comparison to efficient activation by apoA-I^13^, resulting in poor generation of the core lipid of esterified cholesterol and poor capacity of inducing cell cholesterol release by its exchange pathway, and 2) stable binding of apoA-II to HDL particles compared to high exchangeability of apoA-I among HDL and other lipoproteins to make particles more “stable”^14^,^15^. These two types of HDL particles were demonstrated for differences in structure^16^,^17^,^18^,^19^,^20^,^21^,^22^,^23^ and functions^19^,^21^,^22^,^24^. However, information of such HDL subpopulation is still limited, either quantitatively or qualitatively. Most of the observations are descriptive based on defined particles isolated from limited numbers of donors.

We therefore analyzed the data of apoA-I, apoA-II and physical association of apoA-I and apoA-II from more than 300 Japanese subjects previously collected and accumulated in 1994^25^. The results revealed unique feature of HDL containing apoA-I and apoA-II, in comparison to that having apoA-I without apoA-II, and provide important insight on the function of HDL for cholesterol transport in human.

## Materials and Methods

### Study Subjects

Blood samples were collected in 1994 from randomly selected subjects who visited Omiya City Clinic for regular health check-up, 177 males and 137 females, all upon informed consent in fasting state under unlinkable anonymity for individual identification^25^. After clotting, plasma and buffy coat were taken for determination of lipid and apolipoprotein levels, and CETP genotypes, respectively, at National Institute of Nutritional Sciences completed in 1994–1995. Protocol for the operation was retroactively in accordance with Ethical Guidelines for Epidemiological Research first installed in 2002 < https://www.niph.go.jp/wadai/ekigakurinri/guidelines.pdf> and Ethical Guidelines for Medical and Health Research Involving Human Subjects in 2015 < http://www.mhlw.go.jp/file/06-Seisakujouhou-10600000-Daijinkanboukouseikagakuka/0000080278.pdf> by Ministry of Health, Labour and Welfare of Japan. A part of the data of this study was previously published elsewhere^25^ and the current analysis of the data was approved by the Ethics Committee for Human Gene and Genome Research at Ochanomizu University.

### Measurements of lipids and apolipoproteins

Fasting blood plasma was obtained by centrifugation of the blood at 1200 × g for 20 min at 4 °C. Total cholesterol (TC), triglyceride (TG), and HDL-cholesterol (HDL-C) levels in plasma were determined by enzymatic methods by using commercially available assay kits (SEIKEN T-CHO(S), SEIKEN FG-TG(II), SEIKEN HDL-CHO, respectively, DENKA SEIKEN, Ltd, Tokyo) in a biochemical analyzer TBA-60R (TOSHIBA MEDICAL SYSTEMS CORPORATION, Tochigi, Japan). ApoA-I and apoA-II were determined by using commercial immunoturbidimetry assay systems (apoA-I auto · 2, apoA-II auto · 2, Daiichi Pure Chemicals Co., Ltd., Tokyo). All procedures of measurement were done automatically with a biochemical analyzer COBAS MIRA (Roche Diagnostics Corporation, Indianapolis, USA).

### Measurement of apoA-I unassociated with apoA-II by rocket immunoelectrophoresis

ApoA-I unassociated with apoA-II was determined with the HYDRAGEL LPAI PARTICLES Kit (Sebia, Issy-les-Moulineaux, France) by electroimmunodiffusion technique^6^,^26^. The standards and plasma samples (100-fold diluted in saline) were applied into the agarose gel containing anti-apoA-I antibody and excess amount of anti-apoA-II antibody and were electrophoresed at 20 °C, 150 V, for 3 h after diffusing for 20 min. We used our own migration condition as it had been verified to be accurate by intro-, inter-, and linearity-assay. After removing the remaining proteins, the gel was dried and stained with 0.2% acid violet in 10% acetic acid for 5 min. The gel was destained and dried, then the heights of the rockets of apoA-II-unassociated apoA-I were measured and its apoA-I concentration was determined with calibrated standard curve. This value was considered as apoA-I concentration on LpAI. ApoA-I concentration on LpAI:AII was calculated as [total apoA-I]–[apoA-I in LpAI]. The method was also validated with an independent technique by using combination of immunoprecipitation of LpAI:AII with anti-apoA-II antibody and turbidimetric immunoassay with anti apoA-I antibody^27^ for 26 samples randomly chosen (Supplementary Figure).

### HPLC analysis

Plasma sample was treated with anti-apoA-II antibody conjugated with Sepharose 4B gel to adsorb apoA-II-containing lipoprotein particles. The whole plasma and the supernatant after the treatment were analyzed by high performance liquid chromatography as described elsewhere^3^,^28^, by using four tandem TSKgel LipopropakXL columns (Tosoh, Japan) eluted withTSK eluent Lp-1 (Tosoh). Five-microliter plasma sample was analyzed by monitoring cholesterol concentration with an enzymatic assay system. The elution profile of LpAI:AII HDL was calculated as the HDL peak of total plasma minus that of the supernatant by using software for component analysis^29^,^30^.

### CETP genotype determination

In order to differentiate the subjects of normal and modified HDL metabolism, CETP genotype was determined for intron 14 (1452G-A) and exon 15 (D442G) mutations, since the prevalence of these activity-deficient mutations are uniquely high among East Asians including Japanese^31^,^32^,^33^. Fasting blood was collected into a tube containing EDTA-2Na and centrifuged at 1200 × g for 20 min at 4 °C. Genomic DNA was extracted from buffy coat (leukocyte-enriched fraction) with IsoQuick nucleic acid extraction kit (MicroProbe Corp., Bothell, WA, USA). Purified DNA was dissolved in RNase-free water and stored at 4 °C. The DNA was amplified using primers designed for the domain including intron 14 and exon 15 of the CETP gene by polymerase chain reaction (PCR). After verifying amplification of the target DNA fragments of approximately 1400 bp by agarose gel electrophoresis, 1μl of the DNA fragment solution was spotted onto a positively charged nylon membrane (Biodyne^®^ B Nylon Membrane, NIPPON Genetics Co, Ltd., Japan) to detect four genotypes (intron 14 wild/variant and exon 15 wild/variant). The membranes were rinsed with 10 ml of 0.4 M NaOH-1 M NaCl solution for 2 min to denature DNA, dried, rinsed with 10 ml of 0.5 M Tris HCl-1 M NaCl for 2 min twice, dried, and baked in an UV linker to fix DNA. DNA was detected as follows by using a DIG Nucleic Acid Detection Kit (Boehringer Mannheim Biochemica, Germany). The membranes were pre-hybridized in 5 × SSC buffer (0.75 M NaCl, 0.075 M sodium citrate) containing 0.5% blocking reagent, 0.1% N-lauroylsarcosine Na-salt, and 0.02% SDS for an hour at 37 °C. The DNA on the membranes were then hybridized overnight at 37 °C applying digoxigenin labeled and non-labeled with a specific probe. Following hybridization, the membranes were washed twice in 5 × SSC containing 0.1% SDS for 10 min at 37 °C. After washing membranes, Dig-labeled DNA was detected by enzyme-linked immunoassay using anti-digoxigenin alkaline phosphatase conjugate. A subsequent enzyme-catalyzed color reaction using 5-bromo-4-chloro-3-indolyl phosphate and nitroblue tetrazolium salt produced an insoluble blue precipitate that made hybrid molecules visible.

### Simplified Model Analysis for apoA-I Distribution Among HDL Particles

In order to estimate contribution of any specific factor to distribution of apolipoproteins among HDL particles, the data was attempted to fit a simplified statistical probability model as defined below. The model was based on the assumptions; 1) All HDL particles have an equal number of independent “binding site” for helical apolipoproteins, 2) All the “binding sites” on HDL surface are occupied either with apoA-I or apoA-II. 3) Two disulfied-dimer apoA-II molecules displace one apoA-I with absolute priority with no reverse displacement due to high lipid-affinity of apoA-II^14^,^15^. The equations for fitting were derived using the same principle as those derived for apoE binding to microemulsions^34^. When the total number of HDL particle is N, the total number of apoA-II molecule is M and the number of binding site for apoA-II per single HDL particle is B (and therefore that for apoA-I is B/2), probability for a particular HDL particle to be unoccupied with apoA-II, Pe, is calculated as follows. Probability of the first binding site of the first HDL particle free from apoA-II is expressed as 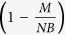, and likewise for the second binding site as 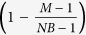, the third as 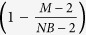 and so on to the last binding site (number B) as 
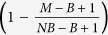
. Therefore,
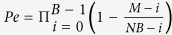
 as for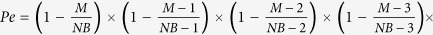 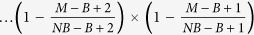. Since N ≫ B and M ≫ B, the equation can be simplified as 
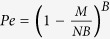
where 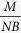 can be considered as an average saturation of the binding site by apoA-II (r). Thus, 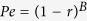. Because it is assumed that all the binding sites unoccupied with apoA-II are occupied with apoA-I, the proportion of apoA-I in apoA-II-unassociated HDL (LpAI)(s) is 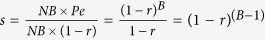. However, if the clearance rate from plasma of LpAI and LpAI:AII is different, a constant factor, c, must be installed. Thus, 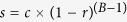. This equation is converted into linearized format of In *s *= (B−1) × In (1−*r*) + In *c*. The value r is to be calculated from apoA-I/apoA-II molar ratio in plasma (R) as 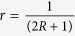 due to the assumption that all the apoA-I and apoA-II molecules are HDL-bound occupying all the “binding sites” and that two apoA-II molecules are equivalent to one apoA-I in the occupancy of the binding sites. Therefore, when the value of r and R are available, the data can be plotted according to this linearized form of the equation, yielding a slope as the number of apoA-II binding site and a Y-axis intercept as a parameter for the relative catabolic rate of two HDL species.

### Statistical analysis

Difference between two groups was analyzed using Unpaired t-test, or Unpaired t-test with Welch’s correction if the variance between the two groups cannot be assumed equal. Difference with p < 0.05 was considered significant.

## Results and Discussion

Basic information of the study subjects is summarized in Table 1. Of the total 314 subjects, 177 were male and 137 were female. Mutations of the CETP gene were identified in 38 subjects; 6 (3 males and 3 females) as heterozygotes of 1452G-A in intron 14, 31 (17 males and 14 females) as heterozygotes of D442G and one male as a homozygote of D442G. HDL-C levels and the related parameters were higher in females than males and in heterozygous CETP deficiency than its wild type. The levels of HDL-C and apoA-I were consistent with other epidemiological data of Japanese^35^. Prevalence of D442G heterozygote was 9.9% and 1452G-A was 1.9%, being consistent with other survey among Japanese and East Asians^31^,^32^,^33^.

**Figure 1:**
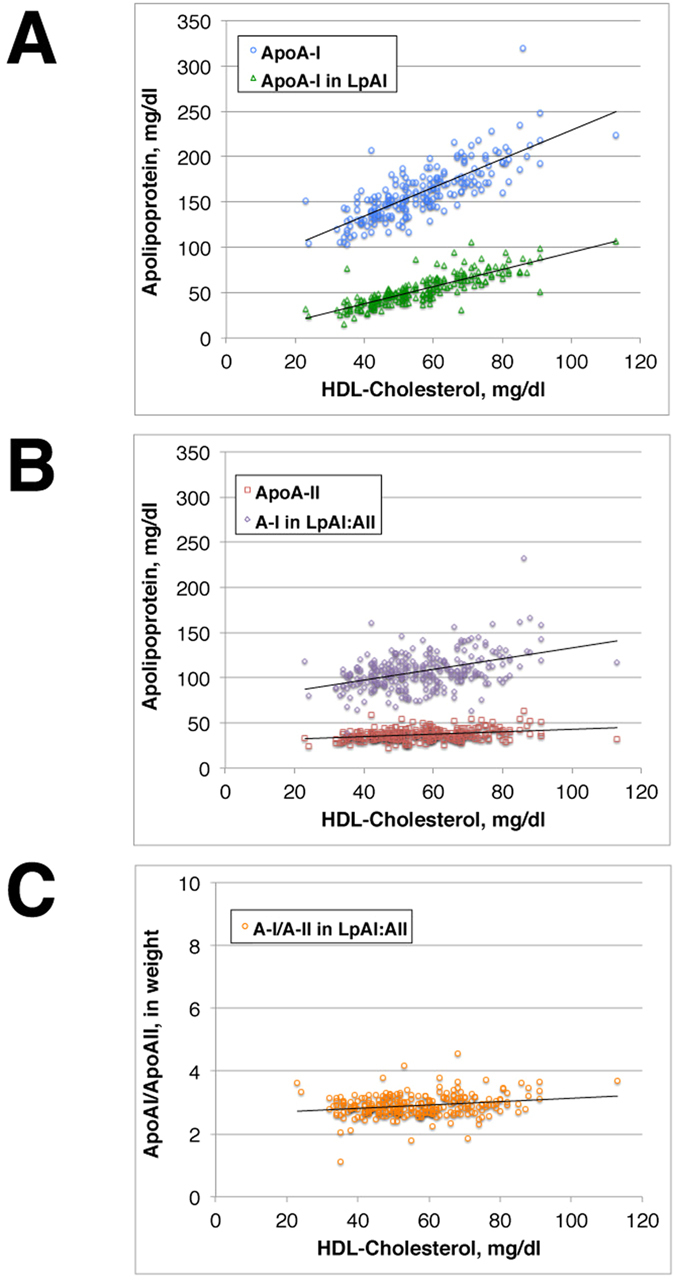
Plots against HDL-cholesterol, of total apoA-I and apoA-I in HDL without apoA-II (LpAI) (**A**), of apoA-II and apoA-I in HDL with apoA-II (LpAI:AII) (**B**), and weight ratio of apoA-I to apoA-II in LpAI:AII (**C**), of the subjects of wild type of CETP genotype.

**Figure 2:**
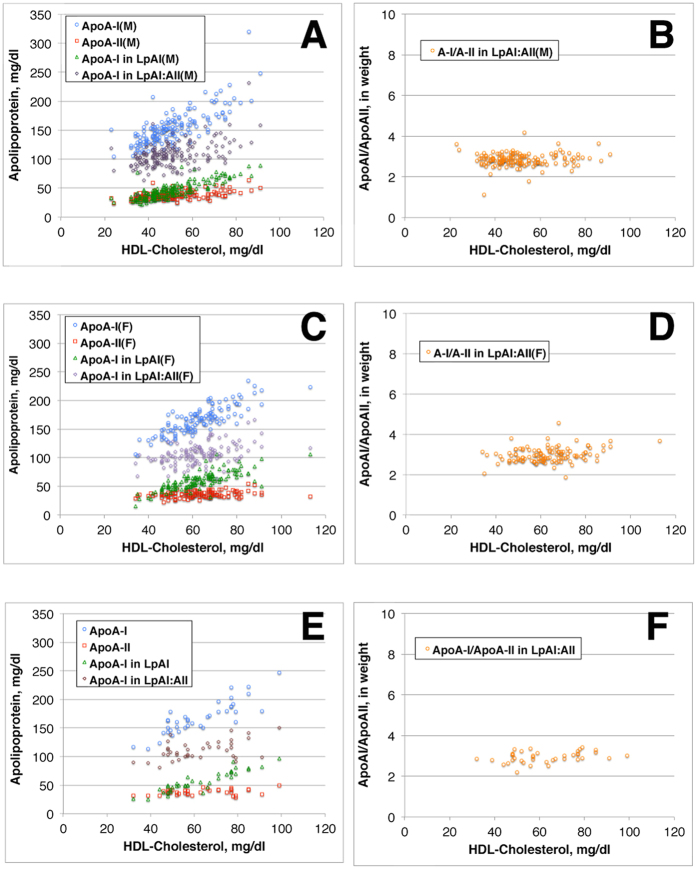
Plots against HDL-cholesterol, of total apoA-I and apoA-I in LpAI, apoA-II and apoA-I in LpAI:AII (**A**), and weight ratio of apoA-I to apoA-II in LpAI:AII (**B**) of the male subjects of wild type of CETP genotype. The same analyses for female subjects of the CETP wild type (**C,D**), and for the male and female subjects of heterozygous CETP mutants (**E,F**).

Figure 1 demonstrates relationship of apoA-I, apoA-II, and apoA-I in LpAI and LpAI:AII to HDL-C of the wild type subjects of CETP genotype. Plasma total ApoA-I increases linearly with HDL-C, and apoA-I in LpAI (apoA-II-free apoA-I) also increased along with HDL-C with a slope somewhat lower than total apoA-I (Panel A). On the other hand, apoA-II also linearly increased but only very slightly and apoA-I in LpAI:AII (apoA-II-associated apoA-I) increased but also only slightly along over the range of HDL-C increase (Panel B). Therefore, the increase of apoA-I along with the increase of HDL-C is largely accounted for by the increase of apoA-I in LpAI. Accordingly, the ratio of apoA-I in LpAI:AII to apoA-II remained constant at around 3 in weight, or 2 in molar ratio throughout the range of HDL-C increase (Panel C). This molar ratio is consistent with most of previous publications based on selected small numbers of samples^17^,^18^,^19^. Figure 2 shows the analyses of wild type samples for males and females separately (Panels A–D). In spite of substantial difference of these parameters between males and females, the relationships of the apolipoprotein parameters to HDL-C were all the same. ApoA-I/apoA-II ratio in LpAI:AII was also same at around 3 in weight and 2 in molar between males and females. The analysis for heterozygous CETP deficiency also yielded same results as the wild type subjects (Panel E, F). ApoA-I in LpAI thus increases by three times from 25 to 75 mg/dl over the increase HDL-C from 30 to 80 mg/dl, while apoA-I in LpAI:AII increased only less than 30%, from 90 to less than 120 mg/dl, over the same range of HDL-C, regardless of sex and CETP genotype.

From the results of the analysis above, it is concluded that human plasma contains constant amount of LpAI:AII particles containing a fixed molar ratio of apoA-I to apoA-II, 2 to 1, regardless of total HDL concentration. Increase of HDL thus predominantly depends on the concentration of LpAI.

**Figure 3:**
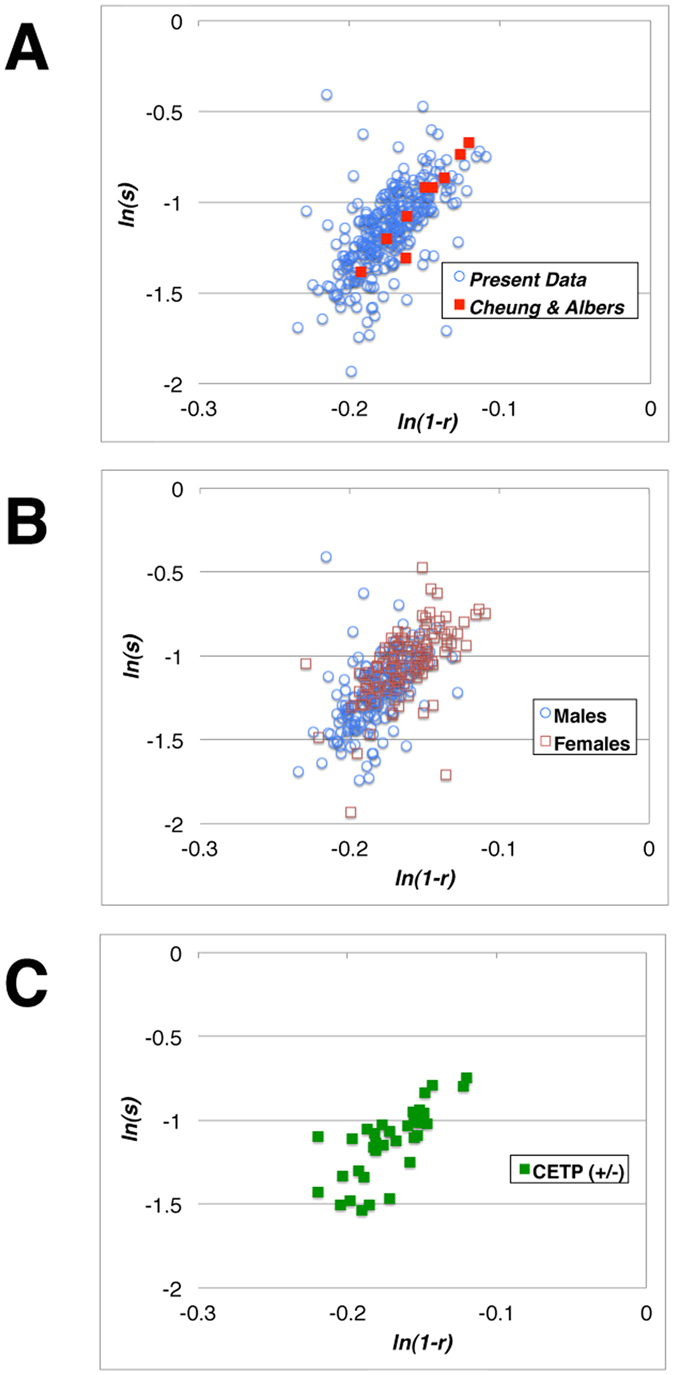
Plots for consistency of LpAI / LpAI:AII distribution with statistical partitioning. Panel A represents the plots for the wild type subjects with respect to CETP genotype of the current study, as well as those from the previous reports by Cheung and Albers^18^. Panel B shows the wild type subjects of the current study, male and female, shown separately. Panel C shows the same plot of the heterozygous CETP mutants. The equation used for the analysis is described in the method section of the text.

It is wondered then whether distribution of apoA-I and apoA-II among HDL particles is regulated merely by statistical probability for occupying binding site as assumed earlier or involves any additional specific factor such as biochemical/biological reactions. The data were thus analyzed according to a model based on statistical distribution of apolipoproteins among “binding sites” of HDL surface^34^. An oversimplified statistical distribution model was applied as described in the method section. The data of the wild type subjects are plotted according to the linearized equation, In *s* = (*B*−1) × In (1−*r*) + In *c*, as defined in the method section, in Fig. 3A. The results taken from a previous publication^18^ were also plotted in the same graph. Both sets showed consistent profiles and gave similar linear fitting in this model. The least square linear regression best fit of the current data gave In *s *= 6.49 × In(1−*r*)−0.019 while the previous sets gave a slope 10.3 and the intercept 0.56. Male and female data gave similar parameters when analyzed separately as In *s *= 5.96 × In (1−*r*)−0.14 and In *s* = 5.82 × In (1−*r*)−0.102, respectively (Fig. 3B). Those of heterozygous CETP deficiency gave In *s* = 6.60 × In(1−*r*)−0.0014 (Fig. 3C). Thus, from the set of the data analyzed here, number of apoA-II per particle (B) can be estimated 7–8 and c value is around 1. Therefore, on the assumption of homogeneous particle size of HDL, the average HDL particle is capable of accommodating four apoA-I molecules or 6–9 of apoA-II. Distribution of apoA-II and apoA-I among the particles is thus not inconsistent with partitioning according to statistical probability.

**Figure 4:**
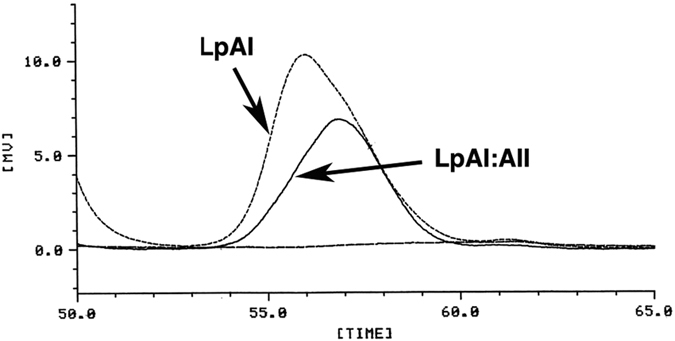
Elution profile of cholesterol of LpAI and LpAI:AII with molecular sieve HPLC. The method is described in detail in the text. The elution profile was monitored with total cholesterol before and after immuno-removal of apoA-II-containing HDL with immobilized anti-apoA-II antibody. The post-treatment profile represented LpAI and LpAI:AII was calculated by subtracting the LpAI profile from that of total HDL.

**Figure 5:**
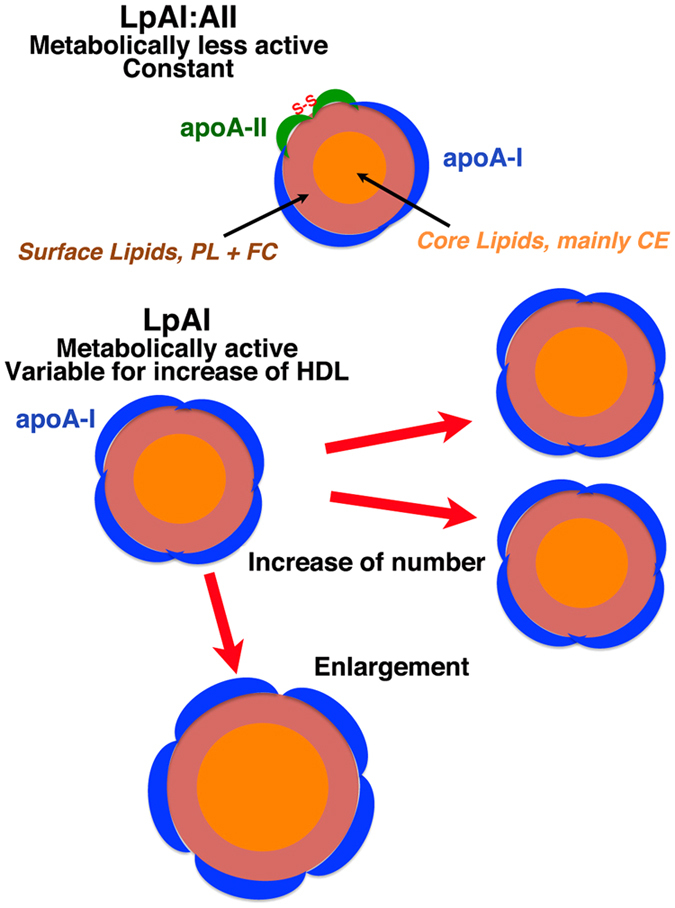
Model for LpAI and LpAI:AII. LpAI:AII particles contain one dimeric apoA-II and two apoA-I molecules, and are stable and perhaps metabolically less active with fixed constant amount. Average LpAI particles accommodate four apoA-I molecules, which are variable to determine total HDL concentration. The increase of HDL may be due to the increase of the number of LpAI or by the increase of HDL size to accommodate more apoA-I molecules. PL, phospholipid; FC, free cholesterol; CE, esterified cholesterol.

Previous reports however indicated that HDL particles containing apoA-II (LpAI:AII) are smaller or heavier than those with apoA-I but without apoA-II (LpAI)^16^,^17^,^19^,^22^,^28^,^36^,^37^. We also confirmed this by HPLC analysis of human HDL, as demonstrated in Fig. 4. The peak of HDL containing apoA-II, LpAI:AII, was smaller than HDL containing apoA-I but not apoA-II, LpAI. The difference of the diameter was estimated as about 20%, consistent with the previous estimation^16^,^19^,^22^,^36^. LpAI:AII was shown to have apoA-I and apoA-II in a molar ratio of 2 (Figs 1 and 2). Assuming average LpAI accommodates four apoA-I molecules, LpAI: AII would fit to contain two apoA-I molecules and one apoA-II molecule because the alternative possibility of four apoA-I and two apoA-II molecules is unlikely. If the number of apolipoprotein binding site is proportional to the surface area of the particle, the diameter of LpAI: AII particles is smaller than average LpAI particles by about 20%, consistent with the measured values. Figure 5 illustrates the concept of this scheme. This model is consistent with volumetric simulation of apolipoprotein stoichiometry in HDL previously proposed by Kézdy and his colleagues^36^ and more recently confirmed by HPLC analysis^30^. The scheme is somewhat inconsistent with the earlier assumption that apolipoprotein “binding site” number on HDL particles is same. The linearity of the plot in Fig. 3 may not be sensitive enough to detect this scale of the difference. This is also shown in Fig. 2 where the slope of apoA-I in LpAI plotted against HDL-C is more or less same between the wild type and the CETP mutants in which increase of HDL-C is generally considered due to the increase of the size of HDL.

We here conclude that human plasma HDL is largely composed of two discrete types with respect to apoA-I/apoA-II composition, HDL having only apoA-I (LpAI) and that containing apoA-I and apoA-II (LpAI:AII). The concentration of the latter particle, containing two apoA-I and one apoA-II molecule(s) and smaller than LpAI, is relatively constant regardless of total plasma HDL concentration. On the other hand, LpAI particles having average number of four apoA-I molecules predominantly regulate plasma HDL concentration.

Distribution of apoA-I and apoA-II among HDL particles and thereby generation of LpAI and LpAI:AII seems thus in principle to be due to statistical probability based on common binding sites and higher affinity of apoA-II as defined in the model. However, once LpAI:AII particles are formed, they seem structurally more stable and unlikely change the size since this HDL maintains the fixed apoA-I/A-II molar ratio of 2. The concentration of this particle stays rather constant regardless of variation of total HDL concentration, so that it should be less active with respect to remodeling. However, significant difference in metabolic fate was not apparent in the analyses employed in this paper. More recent work on clearance or distribution of HDL apoproteins^38^,^39^ also seem consistent with the current hypothesis. It is interesting that relative cholesteryl ester content to unesterified cholesterol is reportedly rather higher in LpAI:AII than LpAI^20^,^22^ though apoA-I is known for more efficient “activation” of LCAT than apoA-II. The conclusions are largely consistent with previous discussion on these types of HDL.

Plasma HDL concentration predominantly depends on the concentration of LpAI, so this particle is a determiner of the risk for cardiovascular diseases. The increase in LpAI can be in their number or size, which could appear as a difference of surface/core ratio of the particles and may be represented by apoA-I/HDL-cholesterol ratio (Fig. 5). However, the slope of the apoA-I versus HDL-cholesterol plot for LpAI in Fig. 3 did not show significant difference among the wild type males and females and even heterozygotes of CETP deficiency. Thus, biochemical markers of HDL may not be sensitive enough to detect subtle change in the particle structure. It is of interest that heterozygotes of CETP deficiency exhibited the same profile as the wild type carriers with respect to structure and behavior of LpAI and LpAI:AII though they generally appear with increased size of HDL. More information is required for the size of the HDL subclasses in these subjects in order to understand roles of CETP in HDL interconversion.

The data here were based upon the rocket immunoelectrophoresis assay for differentiation of total apoA-I and apoA-II-associated apoA-I. The method potentially involves a number of problems as any other immuno-differential methods for the same purposes, such as specificity of the antibody, release of apoA-I from HDL, accuracy of quantitative staining, etc. The method was validated with an independent turbidimetric immunoassay^27^, which largely assured acceptable liability of the rocket immunoelectophoresis method by showing linearity of the relationship between the two method (Supplementary Figure), except for apparently higher background in the reference method perhaps from the background turbidity. Thus, the conclusion here may be verified by using some other methods for differentiation of LpAI and LpAI:AII.

These two subclasses of HDL may largely represent HDL3 and HDL2, but their physiological significance such as functions in cholesterol transport or discriminating risks for any disease is not apparent^6^,^19^,^20^,^22^,^40^,^41^,^42^,^43^. Further analysis of the data accumulated here may reveal any pathophysiological significance and therefore rationale for measuring the subclasses.

## Additional Information

**How to cite this article**: Kido, T. *et al*. Bioinformatic Analysis of Plasma Apolipoproteins A-I and A-II Revealed Unique Features of A-I/A-II HDL Particles in Human Plasma. *Sci. Rep.* **6**, 31532; doi: 10.1038/srep31532 (2016).

## Supplementary Material

Supplementary Information

Supplementary Information

## Figures and Tables

**Table 1:** Characteristics of the study subjects.

	CETP Wild Type			CETP Variants	
	Total	Male	Female	heterozygote	homozygote
	n = 276	n = 156	n = 120	n = 37 (1452 G-A, 3 males, 3 females) (D442G, 17 males, 14 females)	n = 1 (D442G, 1 male)
Age (yrs)	47 ± 7	47 ± 7	47 ± 6	47 ± 6	54
TC (mg/dL)	192 ± 32	194 ± 35	189 ± 29	198 ± 32	227
TG (mg/dL)	125 ± 101	152 ± 124	91 ± 39	108 ± 56	210
HDL-C (mg/dL)	56 ± 14	51 ± 13	63 ± 13**	63 ± 16^§^	48
apoA-I (mg/dL)	159 ± 28	154 ± 29	166 ± 25^††^	168 ± 29	164
apoA-II (mg/dL)	37.0 ± 6.2	37.8 ± 6.6	36.0 ± 5.5^†^	38.0 ± 5.0	37.1
apoA-I in LpAI (mg/dL)	52.3 ± 16.5	46.5 ± 14.5	59.7 ± 15.8**	56.7 ± 18.0	49.9
apoA-I in LpA-I:A-II (mg/dL)	107 ± 20	107 ± 22	106 ± 18	112 ± 17	114.1
apoA-I /apoA-II in LpA-I:A-II	2.90 ± 0.34	2.85 ± 0.32	2.97 ± 0.36*	2.94 ± 0.27	3.08

The data are presented as Mean ± SD. * and ^†^indicate the reults of Unpaired t test and Unpaired t test with Welch’s correction, respectively, from wild type males as **p < 0.001, *p < 0.01, ^††^p < 0.001, and ^†^p < 0.05. ^§^Indicates p < 0.01 from wild type total by the Unpaired t test.
