# Supplementary material for: Bioinformatic Analysis of Plasma Apolipoproteins A-I and A-II Revealed Unique Features of A-I/A-II HDL Particles in Human Plasma
Source: Sci Rep. 2016 Aug 16;6:31532. doi: 10.1038/srep31532 (PMC4985746; doi:10.1038/srep31532)
Supplement: Supplementary Information [file srep31532-s2.doc]

Supplementary materials for;

**Bioinformatic Analysis on Plasma Apolipoproteins A-I and A-II Revealed Unique Features of A-I/A-II HDL Particles in Human Plasma**

**Toshimi Kido^a^, Hideaki Kurata^b^, Kazuo Kondo^c^, Hiroshige Itakura^d^, Mitsuyo Okazaki^e^, Takeyoshi Urata^f^ and Shinji Yokoyama^g^**

^a^Institute of Environmental Science of Human Life, Ochanomizu University, Bunkyo-ku, Tokyo 112-8610, Japan

^b^Division of Diabetes, Metabolism and Endocrinology, Department of Internal Medicine, The Jikei University School of Medicine, Nishi-Shimbashi, Minato-ku, Tokyo, 105-8461, Japan

^c^Department of Food and Nutritional Science, Toyo University, Itakura-machi, Ora-gun, Gunma 374-0193, Japan

^d^Shinagawa East One Medical Clinic, Minato-ku, Tokyo 108-0075, Japan

^e^Tokyo Medical and Dental University, Bunkyo-ku, Tokyo 113-8519, Japan

^f^International Mibyou (Pre Symptomatic Medicine) Medical Center,
Sanuki-chou, Ryugasaki, Ibaraki 301-0033, Japan and Department of Pharmacogenomics, Showa University, Hatanodai, Shinagawa-ku, Tokyo 142-8555, Japan

^g^Nutritional Health Science Research Center, Chubu University, Matsumoto-cho, Kasugai 487-8501, Japan

Correspondence should be addressed to: Shinji Yokoyama at Chubu University by [syokoyam@isc.chubu.ac.jp](mailto:syokoyam@isc.chubu.ac.jp).

The present address of Takeyoshi Urata: Department of Diabetes, Metabolism and Endocrinology, Showa University School of Medicine, Shinagawa-Ku Tokyo 142-8666, Japan

The work was supported in part by a grant from Japan Health Science Foundation and by MEXT-supported Program for the Strategic Research Foundation at Private Universities (S1201007) and by a Grant-in-Aid from MEXT-Japan (15H02903).

Legend for Supplementary Figure

Validation of the method to measure LpAI and LpAI:AII. Randomly chosen 26 samples from the wild type subjects were assayed for LpAI by immunoelectophoresis and the reference method of turbidimetric immunoassay. The antibody was raised against human apoA-II and human apoA-I in goats plasma. Ten microliter sample serum was mixed with the antibody and detergent to make the solution 70 μL to precipitate LpAI:AII and the supernatant 9 μL was diluted with 300 μL buffer and mixed with anti-human apoAI goat antibody to make the final incubation volume 409 μL. Turbidity of the mixture was measured as absorbance at 600 nm as apoA-I unassociated with apoA-II as a parameter for LpAI.

**A.** LpAI measured with turbidometric immunoassay (TIA) versus LpAI measured with immunoelectrophoresis (RKT). **B.** ApoA-I, apoA-II and LpAI:AII measured by TIA and RKT.
